# Wildfire risk perception survey: insights from local communities in Tuscany, Italy

**DOI:** 10.1186/s42408-025-00380-5

**Published:** 2025-06-20

**Authors:** Silvia Calvani, Riccardo Paoloni, Cristiano Foderi, Niccolò Frassinelli, Judith A. Kirschner, Alessio Menini, Glenda Galeotti, Francesco Neri, Enrico Marchi

**Affiliations:** 1 Department of Agricultural Food Environment and Forestry Sciences and Technologies: Università Degli Studi Di Firenze Dipartimento Di Scienze E Tecnologie Agrarie Alimentari Ambientali E Forestali, University of Florence, Via San Bonaventura 13, Florence, 50145 Italy; 2 Institute of Geography, University of Bern: Universitat Bern, Hallerstrasse 12, Bern, 3012 Switzerland; 3 Euro-Mediterranean Center for Climate Change, Fondazione Centro Euro.Mediterraneo Sui Cambiamenti Climatici, Via De Nicola 9, Sassari, 07100 Italy; 4 Department of Land Environment Agriculture and Forestry, University of Padua, Università Degli Studi Di Padova Dipartimento Territorio E Sistemi Agro-Forestali, Viale Dell’Università 16, Legnaro, PD 35020 Italy; 5 Department of Education Languages Intercultural Studies Literature and Psychology: Università Degli Studi Di Firenze Dipartimento Di Formazione Lingue Interculture Letterature E Psicologia, University of Florence, Via Laura 48, Florence, 50121 Italy

**Keywords:** Local communities, Socio-ecological system, Paradigm shift, Prevention, Risk perception, Social learning and participation, Wildfire management, Wildfire risk

## Abstract

**Background:**

Wildfire is a complex chemical, physical, and sociological phenomenon deeply rooted in the historical relationship between humans and fire. Today the wildfire risk is one of the human challenges. Effective management requires collaboration among multiple stakeholders across different levels. The risk perception and vulnerability at the local community level explain why and how individuals consider certain policies or mitigation behaviors. Thus, wildfire risk fits within the framework of socio-ecological systems.

This study focused on four fire-prone areas in Tuscany, Italy, aiming to explore local wildfire risk perception. Risk perception is a social parameter, derived from media habits, memory, history, concerns, and beliefs. Two different surveys were used to consult two groups: experts (e.g., wildfire technicians, policymakers, business activities, and rural associations) and non-experts (e.g., random residents, students, and tourists), then compared to investigate possible gaps. Several questions were asked regarding demographics, relationship with the territory, current management system, relationship with fire and media, risk perception, and others.

Results were compared according to the critical area or the type of respondents, and several analyses were conducted to identify weaknesses, strengths, and areas for improvement to raise awareness and lower the risk.

**Results:**

Findings revealed differences in perception, more between the two groups than across locations, highlighting gaps that need to be addressed. A general underestimation of risk, with an overall optimism, was found in the non-expert group, indicating the need for further qualitative research to understand these aspects better. The interviews suggest public action as the main component to implement change.

**Conclusions:**

The paradigm shift toward prevention represents a core change and challenge. An exchange between scientific and local knowledge is desirable to address many gaps. We propose awareness raising as a possible starting point and to encourage collective actions in line with suggestions from the interviewees. Continuous monitoring and evaluation of response patterns can inform policy adjustments and resource allocation to enhance resilience and response effectiveness in future emergencies. Further research could aim to develop tools to promote a culture of fire and risk and deepen the analysis of risk perception in the most critical areas.

**Supplementary Information:**

The online version contains supplementary material available at 10.1186/s42408-025-00380-5.

## Resumen

**Antecedentes:**

Los fuegos de vegetación son fenómenos químicos, físicos y sociológicos complejos fuertemente enraizados en la relación histórica entre fuegos y humanos. Hoy, el riesgo de estos incendios es uno de los desafíos que enfrentamos los humanos. El manejo efectivo de estos incendios requiere la colaboración de diferentes grupos de interés en varios niveles. La percepción del riesgo y vulnerabilidad a nivel de comunidad local explica el por qué y el cómo los individuos consideran algunas políticas y comportamientos para su mitigación. El riesgo de incendios de vegetación encaja, entonces, dentro del marco de los sistemas socio-ecológicos. Este estudio se enfocó en cuatro áreas proclives a fuegos en la región de Toscana, Italia, con el objetivo de determinar la percepción local del riesgo de incendios de vegetación. Esta “percepción del riesgo” es un parámetro social, derivado de los hábitos de los medios, de la memoria, de la historia, las preocupaciones, y las creencias. Dos relevamientos diferentes fueron usados para consultar a dos grupos: expertos (i. e. Técnicos en manejo del fuego, gestores políticos, personas con actividades económicas, y asociaciones rurales), y no-expertos (i. e. residentes en general, estudiantes, y turistas), y luego los comparamos para detectar posibles diferencias. Diferentes preguntas fueron realizadas relacionadas con la demografía, la relación con el territorio, el manejo actual de los recursos, la relación entre los incendios y los medios, la percepción del riesgo, y otras. Los resultados fueron comparados de acuerdo con el área crítica o el tipo de respondientes, y diversos análisis fueron conducidos para identificar debilidades, fortalezas, y áreas de mejoramiento para aumentar la concientización y bajar el riesgo.

**Resultados:**

Los resultados revelaron diferencias en la percepción, más entre grupos que entre áreas, subrayando vacíos que deben ser adecuadamente atendidos. Una subestimación del riesgo, con un optimismo generalizado, fue encontrado en el grupo de no-expertos, indicando la necesidad de mayores investigaciones cualitativas para entender mejor estos aspectos. Las entrevistas sugieren que las acciones públicas son el componente más importante para implementar cambios.

**Conclusiones:**

El cambio de paradigma hacia la prevención representa el cambio y el desafío central. Un intercambio entre el conocimiento científico y el local es deseable para atender los diferentes vacíos que existen. Proponemos el aumento en la concientización como un posible punto de inicio para llevar a cabo acciones colectivas que estén en línea con las sugerencias propuestas por los entrevistados. El monitoreo y evaluación continua de los patrones de respuestas pueden informar sobre los ajustes de las políticas y el direccionamiento de los recursos para aumentar la resiliencia y la efectividad de las respuestas en futuras emergencias. Investigaciones futuras deberían enfocarse a desarrollar herramientas para promover una cultura de fuegos y riesgo y profundizar así el análisis de la percepción del riesgo en las áreas más críticas.

## Introduction

Wildfires are one of the most important ecological and dynamic disturbance processes, directly or indirectly, globally affecting forests and other landscapes with their related structures and functions (Li et al. 2023). Moreover, wildfires are threatening human infrastructures and people, resulting in fatalities (e.g., premature mortality, suicides), hospitalizations (e.g., respiratory morbidities), as far as affecting mental health and well-being, even miles away from the trigger, due to fire smoke pollution (Johnson and Garcia-Menendez 2022; Molitor et al. 2023; Eisenman and Galway 2022; Gould et al. 2024). The occurrence of increasingly uncontrollable wildfires driven by combined climate and several social and environmental drivers is challenging the management systems (García-Llamas et al. 2019; Essen et al. 2023). Wildfires are now affecting larger areas in Mediterranean Europe, growing in intensity and severity (North et al. 2015; Bowman et al. 2017; Singleton et al. 2019), and changing in time and frequency.

Climate change, including increased heat waves and extended droughts, combined with socioeconomic factors, further exacerbates this process. Indeed, as Pausas and Leverkus (2023) state, the severity of the disturbances depends not only on the intrinsic properties of wildfire (intensity, frequency, magnitude) but also greatly on human aspects (socioeconomic, historical, political, and cultural aspects, i.e., landscape vulnerability). This is particularly relevant in wildland-urban interface areas, where the interconnection between anthropogenic structures, natural spaces, and combustible vegetation is especially tight.

The widespread use of suppression policies in past decades to combat wildfires has proven to be insufficient or problematic, leading to the development of the concepts of the “wildfire paradox” and the “firefighting trap” (Calkin et al. 2013; Ingalsbee 2017; Arévalo and Naranjo-Cigala 2018; Power et al. 2018; Ascoli et al. 2023). As a result, there is now a recognized need to shift towards prevention policies (Bowmann et al. 2011, Moreira et al. 2020, Otero et al. 2017, Pyne 2007, Uyttewaal et al. 2023). Moreover, great social changes during the last century, such as the Industrial Revolution, led to a cascade of impacts both at the local and global levels. Land use change caused rural land abandonment, leading to fuel accumulation, while private ownership caused land fragmentation. The management of wildfires is often tied to the local knowledge of rural communities, developed through a dynamic interaction with the ecological characteristics of the landscape. This knowledge constitutes a fundamental element of the anthropological identity of these communities, shaping their relationship with the environment and their ability to manage the territory. However, the progressive depopulation of rural areas is eroding these knowledge systems, creating a cycle of vulnerability and loss. These factors have particularly complicated forest management near the wildland-urban interface (Aguilar and Montiel 2011; San-Miguel-Ayanz et al. 2013). An additional factor of increasing risk is a counter-trend that happened in the last decades: many people moved back to the countryside for residential purposes, in the so-called Wildland Urban Interface (WUI) (Galiana-Martin et al. 2011; Salis et al. 2014; Àgueda et al. 2023). These new residents often bring with them different perspectives on forests and the ecosystem services they provide, including urban-centric views that prioritize recreational and scenic uses of the forest over more active management practices (Uyttewaal et al. 2023) These factors, combined with the fire exclusion paradigm, contribute to the intensification of wildfire severity, further exacerbated under extreme weather conditions and by increasing risks anticipated due to climate change (Jolly et al. 2015; Parisien et al. 2020).

Until the early 2000 s, wildfire research predominantly focused on physical parameters and environmental drivers. However, several studies have since highlighted the significant role of social dimensions in altering ecosystem dynamics, thus the multi-dimensional nature of the wildfire issue (Pyne 2007; McCaffrey et al. 2013; Moritz et al. 2014; Christianson 2015; Scheller et al. 2019). This approach emphasizes the importance of sociocultural implications in understanding the causes and responses to wildfires, both before and after their occurrence (Castelló and Montagut 2019; Vigna et al. 2021a; Uyttewaal et al. 2023). The human factor influences various wildfire-related processes, such as managing phases, damage mitigation, fatalities, risk reduction practices, and the promotion of prevention and adaptation strategies (Tedim et al. 2018). Humans also shape fire regimes through fuel load alterations, continuity, and microclimates, within complex ecological, economic, and social relationships (Laris 2011). At the same time, fire regimes influence and guide human actions, often reinforcing specific intervention strategies. This interplay calls for incorporating interdisciplinary concepts such as historical ecology (Coughlan and Petty 2012; Coughlan and Petty 2013) and the pyric transition (Pyne 2001). The concept of cultural pyrogeography further explores how human practices interact with vegetative fuel, affecting wildfire frequency and intensity, particularly in the wildland–urban interface (Roos et al. 2014; Wunder et al. 2021).

At the core of such a perspective are the interactions between hazards and humans, starting from people’s perceptions (McCaffrey 2004). Engaging local communities and stakeholders has become critical to raising awareness and fostering effective mitigation and adaptation strategies. This involvement enables communities to inhabit and manage their territories while fostering dialogue and shared understanding (Gordon et al. 2012; Christianson et al. 2013; Slavkovikj et al. 2014; Otero et al. 2018; Meldrum et al. 2019; Ascoli et al. 2023; Palaiologou et al. 2021). The limitations of focusing solely on environmental drivers and the consequences of fire exclusion have driven a paradigm shift in fire research and management (Pyne and Goldammer 1997; Silva and Batalha 2010; Bowman et al. 2011; Eloy et al. 2019; Leone et al. 2020; Moreira et al. 2020). This shift aims to redefine the historical nexus between human societies, rural landscapes, and fire, a relationship long studied in fire ecology (Leone 1996; Daniel et al. 2007). A central challenge in managing the wildfire phenomenon lies in incorporating landscape values, local knowledge, and social perceptions of risk into operational decision-making systems (Otero et al. 2018).

Understanding community perceptions and responses to fire risk offers insights into their relationship with fire, shaped by memories, experiences, and interactions with the environment, other actors, and media (Sousa et al. 2022). Within a social-ecological system framework, both social and biophysical factors contribute to defining risk and its perception, providing a holistic approach to wildfire research (Ager et al. 2015; Tedim et al. 2016; Otero and Nielsen 2017; Kleindl et al. 2018; Hamilton et al. 2019).

This study aimed to investigate the wildfire risk perception in four fire-prone municipalities characterized by gaps. The aim of this study was to investigate the perception of wildfire risk in four fire-prone communities characterized by gaps. The gaps are related to the values we observed in an equation where the wildfire impact index (calculated considering the frequency and extent of forest fires) was divided by the media habits index (based on media flows between authorities and local communities). If the two indices showed large differences, we defined the area as critical (e.g., high communication rates and low wildfire activity, several wildfires and few media talking about them). (Calvani et al. unpublished data). Surveys and talks were addressed to local communities and technical experts (Raftoyannis et al. 2014; Koksal et al. 2019; Sapiains et al. 2020; Hall et al. 2022). The actors involved were chosen after a study of the local contexts. Sociologists, anthropologists, and researchers in human science were also engaged in defining study boundaries and developing participatory pathways (Razzoli 2011; Malfatti 2012). The results were analyzed to understand the level of perception. The final objective of this research was to investigate the perception and thus understand the awareness of people who live in fire-prone territories, as well as their availability in managing the landscape, whether they are technicians or inhabitants, plus possible gaps to fill (Brummel et al. 2010; Meldrum et al. 2015; Schultz et al. 2019; McIntyre and Schultz 2020). The future use of this study will stand in the mitigation strategies and preventive plans that will be developed in these areas by the competent bodies (Champ et al. 2013; Meldrum et al. 2019; Metlen et al. 2021), through the development of participatory pathways. A fundamental long-term goal is the development of a culture of risk, intended as a set of knowledge, skills, and abilities to assess, understand, and manage hazards and related risks caused by particular phenomena and events, as masterfully argued by Pyne in his works (1997, 2001, 2007) or, more recently, by Ottolini et al. (2024).

## Methods

### Background: a context of the areas

**Fig. 1:**
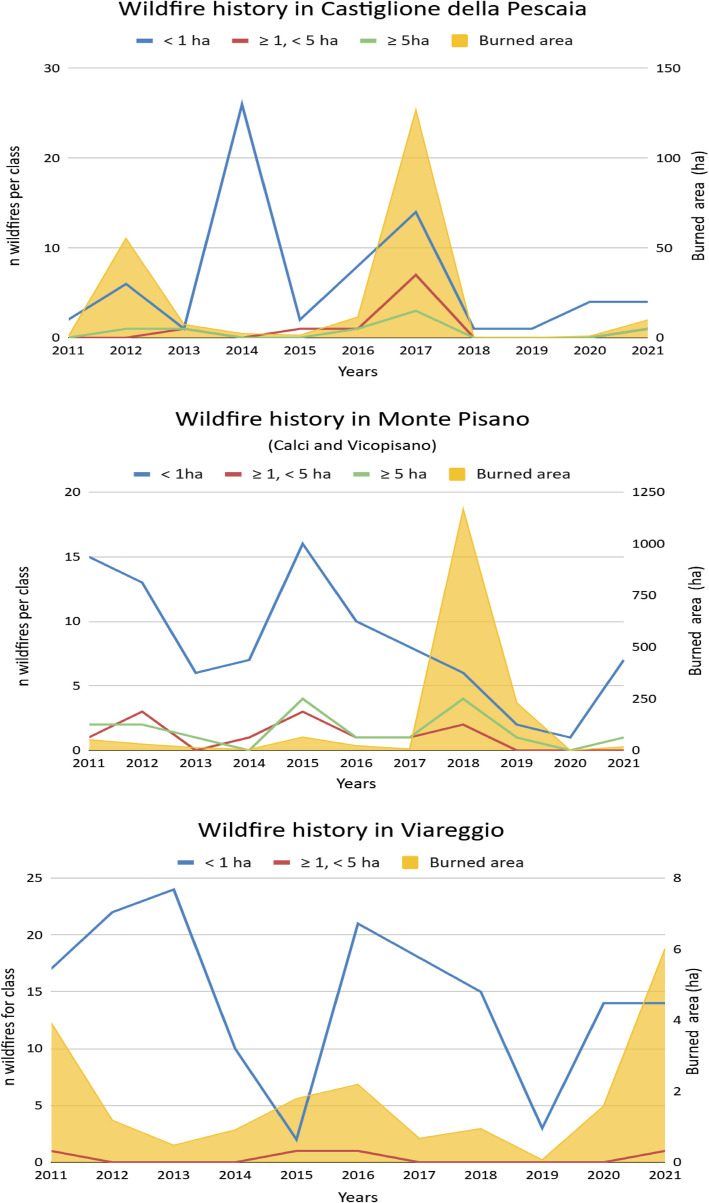
Wildfire history (2011–2021) in each area of study, considering Calci and Vicopisano as one. Comparison of the number of wildfires and the burned area (ha) per year. Viareggio misses data for wildfires with burned area ≥ 5 ha. The wildfire classes used in the Regional Fire Prevention plan (Piano AIB Regione Toscana 2023–2025) were applied

The study focused on four areas of the Tuscany region, in Italy, where low-elevation conifer stands, particularly in coastal areas, are frequently affected by wildfires due to natural predisposition (fire-adapted species) (Lecina-Diaz et al. 2014) or to the absence of preventive interventions. Over past decades, Tuscany has been the site of memorable wildfire events, including incidents in the Polveriera area in 2012 (Massa Marittima, GR), in Montale (PT) in 2017, and more recently on Monte Pisano (PI) in 2018 and Massarosa (LU) in 2022 (Tuscany Region database, https://www502.regione.toscana.it/geoscopio/incendiboschivi.html, February 2023). These wildfires were characterized by difficulties in managing operations, partly due to very high energy levels (i.e., extreme wildfires). Despite the well-established wildfire-fighting organization in Tuscany, extreme events have resulted in considerable damage. Responses from the territory included the formation of so-called fire-wise communities, a risk management tool aiming to reduce the risk at the structural level (e.g., buildings, infrastructures, etc.) down to the community level (e.g., education, raising awareness, natural resource use, etc.), for example, through the establishment of defensible spaces (Tedim et al. 2016). Therefore, we identified four critical areas as suitable for analyzing wildfire risk perception, based on previous research on wildfire media habits and occurrence (Calvani et al. unpublished data), i.e., Calci (PI), Vicopisano (PI), Castiglione della Pescaia (GR), and Viareggio (LU). For consistency with the object of the analyses, we analyzed Calci and Vicopisano as one (i.e., Monte Pisano ridge) for the context analysis and the fire history (Fig. 1). We also conducted a context analysis for each area to retrieve basic information about the targeted sample and to guide the questionnaire building (see Additional files).

### Representative random sampling

We defined the boundary of the sampling frame, i.e., the starting population, as all the people who live or usually visit the critical areas. The sampling was hybrid, applying non-probability techniques and random sampling (Frankel 1987; Neuman 2014). We identified two categories in the investigated population, covering several kinds of local actors (Luu et al. 2024): the group of “non-experts” includes people not involved in activities related to forestry and/or wildfire (e.g., citizens, tourists, students, workers), and the group of “experts” that includes people working in the field of forestry and/or wildfire (e.g., technicians, AIB volunteers, policymakers, environmental associations, business activities in tourism or forest management, parks authorities). According to Reed (https://www.fasttrackimpact.com/post/2019/03/11/how-to-do-stakeholder-analysis, 2019), the former can be considered the marginalized, i.e., those who have limited interest and low influence on management decisions, but who can gain major benefits from this research: we randomly reached them in several circumstances, while varying their gender and age as much as possible. The experts are those whom Reed would define as gatekeepers, so they are actors who could strongly influence the research but are sometimes difficult to reach. We scheduled appointments or corresponded via email, thus applying a convenience method (see Additional files).

### Questionnaire building and administration

After the sample identification, we used anonymous questionnaires to explore wildfire risk perception. The questionnaire included both open-ended and closed-ended questions. Six thematic sections were set up. One questionnaire was built for each group respectively, to target the specific sample better (see Additional files). We organized pilot testing (*n* = 40) with wildfire technicians and random participants to ensure clarity and measure the time required for compilation. Once this phase was completed and the questionnaires checked, the distribution was finally carried out. We spread the questionnaires online via (Google Forms) (over 6 months, April–September 2022) and in person (for 2 months, May–June 2022). We reached non-experts at local public events and other gathering spaces (parks, markets, pools, etc.), through thematically relevant mailing lists (two universities, one forest community) and social networks (local (Facebook) groups, (Instagram) public and personal accounts, (LinkedIn) researchers’ account). Experts were contacted directly, by email, or by scheduling meetings. For both, we produced flyers and posters with QR codes and hung them up in randomly crowded areas as well as in stakeholders’ offices and workplaces.

### Questionnaire analysis

Once the survey closed, we started the dataset analysis. We analyzed the thematic sections (TS) of each questionnaire using different methods, as explained in Table 1. We applied descriptive and statistical methods. We also made a comparison between non-experts’ and experts’ responses, while in other cases, the matching concerned the critical area of belonging.

**Table 1:** In this table, the name of each thematic section is associated with the methods used for the analysis. Six thematic sections of the questionnaire allowed us to compare wildfire risk perception between “non-experts” and “experts”

	Non-experts		Experts	
	Thematic section (TS)	Method	Thematic section (TS)	Method
TS 1	Sample characteristics	Welch’s *t*-test; descriptive statistics	Sample characteristics	Welch’s *t*-test; descriptive statistics
TS 2	Relationship with the territory	Descriptive statistics; Fisher’s exact test	Current wildfire risk management system	Descriptive statistics; Fisher’s exact test
TS 3	Relation with fires and wildfires	Historical memory analysis; word clouds; sentiment analysis; descriptive statistics; Non-parametric tests	Coordination and cooperation in the territory	Descriptive statistics
TS 4	Relation with media	Descriptive statistics; comparison with media habits; Student’s *t*-test/FET	Relation with media	Descriptive statistics; comparison with media habits; Student’s *t*-test/FET
TS 5	Risk perception	Descriptive analysis; Tukey test; FET; *χ*^2^; Kruskal–Wallis	Risk perception	Descriptive analysis; Tukey test; FET; *χ*^2^; Kruskal–Wallis
TS 6	Further remarks	Coding answers; descriptive statistics; FET; Kruskal–Wallis	Further remarks	Coding answers; descriptive statistics; FET; Kruskal–Wallis

The last question of thematic Sect. 6, “Write down some ideas on how to raise awareness on forest fire risk,” required the application of a systematic coding to the answers, sorting them into three categories (Public policies and activities, Fuel management techniques and Other risk prevention practices and tools) and twenty subcategories, thus analyzed according to the group of respondents and the area of belonging (Saldaña Johnny 2015). We finally applied statistical methods through R software (R Core Team, 2022).

## Results

We sent out 458 questionnaires in total, via email and live compilation, receiving 186 responses, with an overall response rate of almost 41%. Results are presented for each thematic section (TS).

### TS 1: Sample characteristics and representativeness

The demographic data of our sample were compared to the real population of the four investigated municipalities (ISTAT https://www.istat.it, January 2024) to check for significant differences. Given the nature of the data (averages in percentage), their non-normal distribution, and the small sample size, the Welch’s *t*-test was computed (Delacre et al. 2017). Age (*t* = − 0.181, df = 5.952, *P* = 0.863), Gender (*t* = − 0.001, df = 4, *P* = 0.999), and Education Level (*t* = 0.181, df = 5.952, *P* = 0.863): in any case, there were no significant differences between the compared groups, thus the representativity of the sample was verified for these characteristics. Finally, descriptive statistics were applied to the sample characteristics (see Table 2). Survey participants indicated mixed employment types, with the general public and private sectors mentioned most frequently next to the agriculture and environmental sectors (firefighting, especially), followed by students and researchers. Across the four sample municipalities, most respondents referred to the Monte Pisano area (40% Calci and 28% Vicopisano).

**Table 2:** Sample characteristics and representativity. Descriptive analysis of the sample, considering the two groups of respondents together (*n* = 186). Data were collected in Tuscany, IT, between April and September 2022

Characteristic	Response categories	% of respondents
*Age*	15–64	82.3
	≥ 65	17.7
	Average	43.2
*Gender*	F	40.1
	M	57.2
	Other	2.7
*Educational degree*	Elementary	1.1
	Secondary	21
	High school	29.6
	Bachelor/master/PhD	48.4
*Employment*	Institutional bodies	24.2
	Companies and private	27.5
	Agriculture/environment/firefight	16.9
	Student/researcher	15.7
	Tourism, culture	3.9
	Retired	8.9
	Unemployed	2.8
*Critical area*	Calci	40
	Castiglione della Pescaia	14.4
	Viareggio	17.8
	Vicopisano	27.8
*Current living location*	Urban center	63.4
	Rural area	36.6
*Past living location*	Urban center	69.4
	Rural area	30.6

Incorrect perceptions of measured trends were found, such as the answer to the question “In your opinion, the Italian forest area is increasing, decreasing or stable?” where 72% chose “decreasing,” the opposite of the real dynamic (37% of the national territory is wooden, with an increase of + 18.4% from 2008 to 2018, RAFT 2017–2018). According to age, some differences are appreciable. 82% of the participants under 49 years of age assessed a decreasing status of the forests. On the other hand, older participants (over 50 years old) chose the decrease in 51% of the cases (Raft 2016).

### TS 2: Relationship with the territory/current wildfire risk management system

Non-experts and experts expressed quite different opinions (FET: *P* ≤ 0.001) when asked about their opinion on prevention measures that are currently in place. On average, non-experts (Q19) were more likely to believe that prevention measures were insufficient (57% of non-experts’ responses) while experts (Q11) mentioned insufficient prevention measures less often (40% of technician responses). Results from Q13, addressed to experts, reinforced this: 70% of respondents stated they worked in the field of prevention and maintenance, which suggests that their perspective is particularly well informed. The only area where a coincidence between experts and non-experts was observed was Viareggio, where an insufficiency in prevention measures was highly noted by both groups of respondents (85% experts, 60% non-experts) and much more strongly than in the other areas.

Concerning wildfire fighting emergency response, on the other hand, there were similar responses between non-experts and experts, who evaluated the current conditions rather positively: experts (Q12) expressed positive opinions by more than 62%, while non-experts (Q18) by almost 72% (FET gave *P* = 0.02).

As for obstacles to fire risk reduction, many experts (74%) found it in the misbehaviors held by people (Q14). Prevention measures currently present in the areas were also questioned by experts and listed, as well as shortcomings rooted in the general system’s societal context (i.e., rural land abandonment, poor information for citizens, fragmentation due to private ownership, and lack of maintenance) (Q11). The critical situation in Viareggio is also explained by mentions such as lack of interest from the agencies, little information for the population, absence of prevention and maintenance, uncontrolled tourism, and abandonment of rural areas, even if the first Specific Plans for Wildfire Prevention and Management (in Italian: PSP—Piani Specifici di Prevenzione AIB, introduced by the Regional Law 99/00) were initiated by D.R.E.A.M. Italia and the Tuscany Region (D.R.E.A.M (2020)).

### TS 3 (non-experts): Relationship with fire and wildfires

There are two different terms for the concept of fire in the Italian language. The word *fuoco* is normally used to indicate a controlled fire event and refers to the effect of combustion manifested in the flame (Treccani Italian encyclopedia), thus to the natural element. The word *incendio*, on the other hand, indicates an uncontrolled fire, i.e., a fire with susceptibility to spreading over wooded, brushy, or arboreal areas (Italian National Law 353/2000), that may become a disaster in case of a vulnerable area. Given these differences, we wanted to investigate the perception of the two terms (from now on: in this section *fuoco* is a fire in English, and *incendio* is a wildfire in English).

**Fig. 2:**
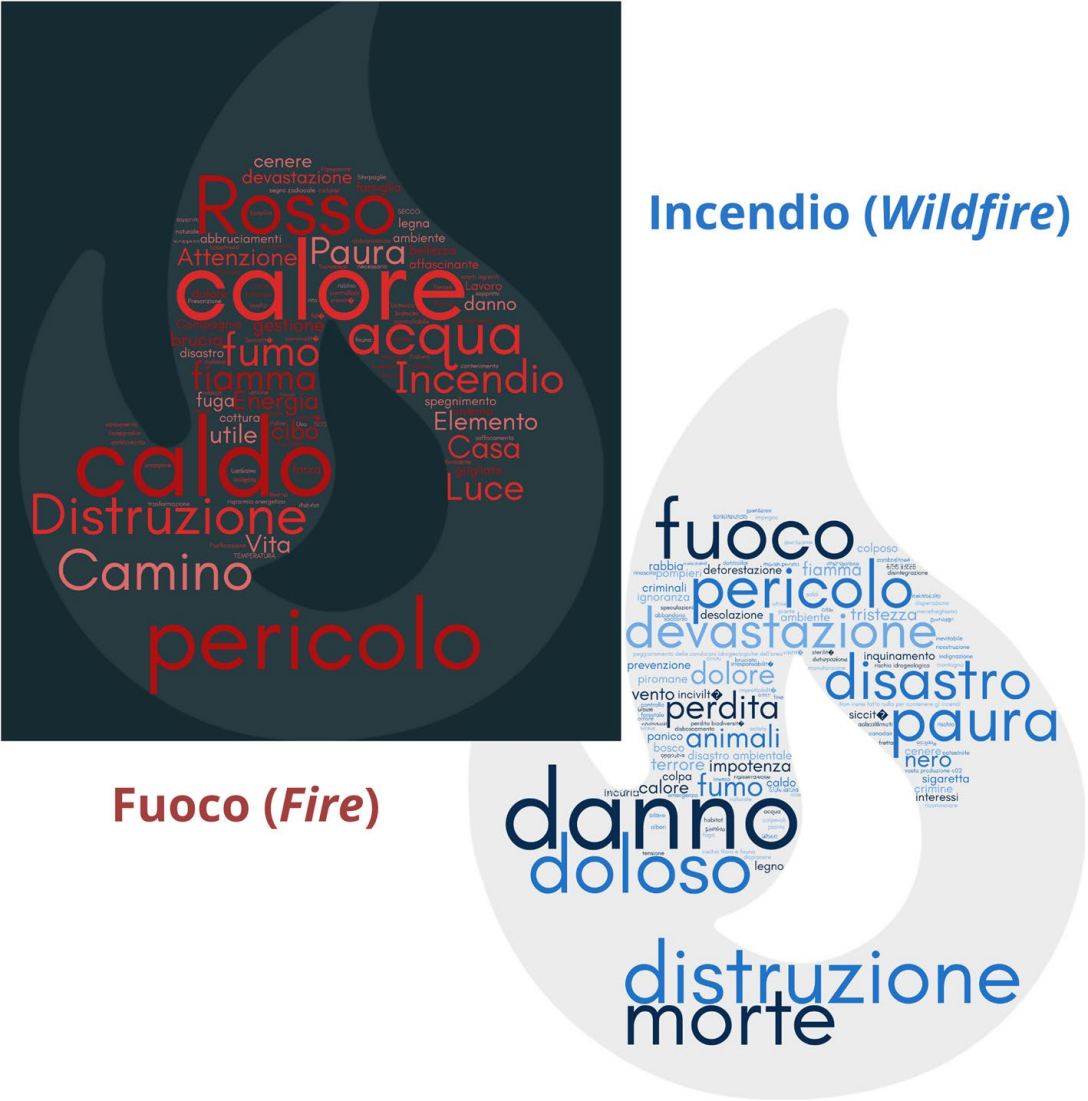
Relationship with fire and wildfires. Word clouds show the terms non-experts used to describe the concept of *incendio* (wildfire, in blue) and *fuoco* (fire, in red). The character dimension is proportionate to the number of repetitions. The most frequent are here translated: Calore and Caldo = Heat; Distruzione = Destruction; Pericolo = Danger; Danno = Damage; Rosso = Red; Morte = Death; Acqua = Water; Doloso = Intentional; Camino = Fireplace; Devastazione = Devastation; Fumo = Smoke; Paura = Fear; Disastro = Disaster; Fiamma = Flame; Perdita = Loss

Considering only the non-experts’ category, fire and wildfire were perceived differently by 78%. The municipality of Calci scored the highest value. From the free word association (Freud 1901) (Q20 and Q21), the IRaMuTeQ software (version 0.8, Pierre Ratinaud, Lerasse Laboratory, Toulouse, France) (Ratinaud, 2009) was used to generate word clouds based on responses to two open-ended questions asking participants to associate three words with *fuoco* and three words with *incendio* (Fig. 2). The two word clouds clearly illustrate the dual perception of fire: while *incendio* evokes terms like “destruction,” “death,” and “danger,” highlighting its dramatic and destructive nature, *fuoco* is associated with words such as “warmth,” “energy,” “home,” and “hearth,” reflecting a more positive and domestic dimension. Experts were asked if, in their opinion, a difference between these words exists in common imagination (Q23): 61% of them felt a distinction, a validation for the previous result.

As for the historical memory analysis (Q23, Q24), data were combined in Table 3 for each critical area. The period considered was 2011–2021, to be consistent with the whole study approach (Calvani et al., unpublished data). In the Monte Pisano area (Calci and Vicopisano) all respondents cited at least one wildfire that occurred during the considered period. 111 events were cited overall. The highest response (61%, *n* = 55) was referred to in 2018, and to the large Calci wildfire (1092 ha). Events that occurred in 2021 were remembered by 18% of non-experts (*n* = 16). Furthermore, someone recalled the Poggio Staffo (Castiglione della Pescaia) wildfire in 2017. In Castiglione della Pescaia, the total number of experienced wildfires was between 2014 and 2017 (58% *Confirmed, where Confirmed means both the place and year of the wildfire cited matched with the regional database*). Notably, 18% of respondents were unaware of any wildfires, even during significant events between 2018 and 2021. Additionally, 17% expressed uncertainty, and 25% provided unmatched references, likely due to the transient nature of populations such as tourists. The Viareggio area had the fewest number of cited episodes (*n* = 5). The totality of the real events concerned 2017, 2018, and 2021 (50% *Confirmed*). Regarding the break from 2011 to 2015, there was no memory of occurrences in the area despite a large number of wildfires (*n* = 77).

**Table 3:** Relationship with fire and wildfires. The historical memory of wildfire for the period 2011–2021 for each area. Comparison across localities on fire history, data surveyed and from the official database. *Confirmed* means both the place and year of the wildfire cited matched with the regional database. In bold are responses with a higher relevance, according to burned area and number of occurrences. Data were collected in Tuscany, IT, between April and September 2022

	Monte Pisano			Castiglione della Pescaia			Viareggio		
Year	Confirmed (83%)	Total burned area (ha)	*N* of total wildfires	Confirmed (58,3%)	Total burned area (ha)	*N* of total wildfires	Confirmed (50%)	Total burned area (ha)	*N* of total wildfires
*2011*	**1**	**51.9**	**18**	0	0.4	2	**0**	**0.22**	**18**
*2012*	**1**	**30.7**	**19**	0	55.4	7	**0**	**0.05**	**22**
*2013*	1	13.3	7	0	7.4	3	**0**	**0.02**	**24**
*2014*	1	4.5	8	**1**	**2.4**	**26**	0	0.09	10
*2015*	**1**	**64.4**	**23**	1	1.4	3	0	0.60	3
*2016*	1	23.1	12	**3**	**11.6**	**10**	**0**	**0.10**	**22**
*2017*	2	7.4	10	**2**	**126.9**	**24**	**1**	**0.04**	**18**
*2018*	**55**	**1167.8**	**12**	0	0.3	1	**2**	**0.06**	**15**
*2019*	7	230.4	3	0	0.2	1	0	0.02	3
*2020*^*a*^	0	0	1	0	0.9	4	0	0.11	14
*2021*	**16**	**17.8**	**8**	0	9.9	6	**2**	**0.40**	**15**

^a^Missing data: actual year of handover of forest fire investigation competencies to the Carabinieri Forestali (L.D. 177/2016)

Considering all areas, 129 wildfires were cited (73% *Confirmed*), with the Monte Pisano area at the top of the rank (83% *Confirmed*). The most mentioned wildfire event, in the last 10 years, was the one of 2018 in Calci with 50 citations. The period with the least citations was 2011–2016 (*n* = 10).

**Fig. 3:**
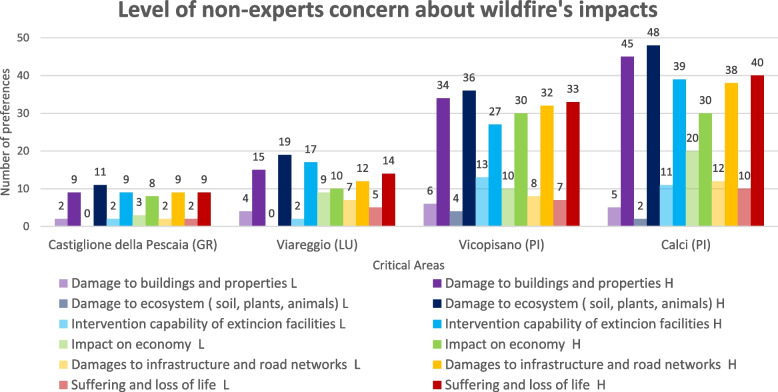
Relationship with fire and wildfires. Level (number of preferences) of non-experts’ concern about these 6 issues: damage to buildings and properties, damage to the ecosystem, the capability of extinction, impact on the economy, damage to infrastructures and road networks, suffering and loss of lives. Each area is represented, and two levels of concern are shown, high (H, in darker colors) and low (L, in lighter colors), for every impact

When asked about their concerns during a wildfire event (Q25), 82% of non-experts felt very concerned about the damage great wildfires could do to the ecosystem (soil, plants, animals): at the top of the rank were Calci and Vicopisano (84%), followed by Viareggio (79%) and Castiglione della Pescaia (73%). In the municipalities of Calci, Vicopisano, and Castiglione della Pescaia, there was on average much worry about damage to buildings and property (48%) and also much concern about possible damage to infrastructure and road networks in Castiglione della Pescaia (55%). The lowest concern was regarding the potential economic impacts of wildfires, without significant differences across areas (Kruskal–Wallis *χ*^2^ = 0.38, df = 3, *P* = 0.95). Among non-experts, 32% expressed little concern, with Viareggio reaching 42%, highlighting a low perception despite several tourism-dependent economic activities in fire-prone areas. Regarding the ability of wildfire fighting to succeed, there was plenty of concern, with 78% (very and fairly) of the total, underlining the importance given to the extinction phase by the non-experts’ group. Results as a number of preferences are shown in Fig. 3, where low and high levels of concern are compared for each issue.

### TS 3 (experts): Coordination and cooperation in the territory

Almost 80% of interviewed experts believed that associations and organizations in their area operated in a coordinated manner (Q24). The highest response was for Calci (96%) and Vicopisano (83%) where the following organizations and associations were mentioned: CVT (Coordinamento Regionale Volontariato Antincendi Boschivi della Toscana (*Regional Coordination of Volunteer Forest firefighters of Tuscany*)), Coordinamento del Monte Pisano (*Monte Pisano Coordination*) and the Comunità del Bosco Monte Pisano (*Monte Pisano Firewise Community*). In the Viareggio area, 31% of experts stated they “Do not know” about a local coordination system nor carry out shared projects with other institutions (46%), both worrying results as regards knowledge and thus risk perception. Indeed, a run-in forest fire fighting and prevention system exists at least at the regional level.

The relationship between experts and citizens (Q27) was rated positively by 59% of total experts: positive judgments (“Good”) were expressed in the areas of Calci (54%) and Castiglione della Pescaia (73%). On the other hand, in Viareggio, most experts (69%) evaluated this relation as “Poor,” justifying the generally low level of knowledge and awareness through the poor interaction among local actors. To improve activities, change proposals were also asked (Q28): training (54% on average, outlier Vicopisano with 67%), financial incentives (45% on average, Viareggio the outlier with 23%), and increased coordination among agencies (45% on average, outlier Vicopisano with 25%), closing with the need for improving the technical support to residents, voted by 58% in Vicopisano and 46% in Viareggio.

### TS 4: Relationship with the media

A comparison was made between the two categories of respondents (experts and non-experts) on the information they had received or given about forest fires (Q15 and Q27). No significant differences were detected among the four areas (*χ*^2^ = 157.68, df = 12, *P* ≤ 0.001), but obtained percentages suggested that the general evaluation stood between “Poor” and “Good” ratings for both (experts 79%, non-experts 89%). Statistical tests were run to understand if non-experts’ and experts’ satisfaction was really at the same level: significant differences were found among the localities and the two groups as for “Poor,” “Good,” and “Optimal” judgments. Viareggio usually stood out: more experts (85%) than non-experts (68%) rated the level as “Poor,” another remark for a low preparation in the area, consistent with other results in previous sections. An analysis of responses to questions Q28–Q30 (for non-experts) and Q16 (for experts) revealed a general trend of more negative experiences reported by non-experts compared to experts (Table 4). This difference is statistically significant (*χ*^2^ = 24.9, df = 12, *P* = 0.015). The largest gap was observed in preventive communication, where 32% of non-experts reported a lack of this tool, compared to only 9% of experts. Additionally, the two most commonly used sources of information (Q31) for non-experts are social networks and newspapers, with 72% of them relying on these channels for information.

**Table 4:** Relationship with media. Evaluation of information received and given, according to each phase of the wildfire management plan, for both interviewed groups. (None: 0 data, Poor: ≤ 30 data in 10 years, Good: ≥ 30 data in 10 years, Optimal: more than 100 data in 10 years). In bold are responses with a higher percentage and interest. Data were collected in Tuscany, IT, between April and September 2022

Experts (*n* = 66)	Prevention	Alert	Emergency	Burned area mgmt	Non-experts (*n* = 120)	Prevention	Alert	Emergency	Burned area mgmt
*% None*	9	2	2	8	*None*	**32**	18	23	**32**
*% Poor*	**39**	24	21	24	*Poor*	**38**	28	**47**	**38**
*% Good*	**39**	**38**	**42**	**42**	*Good*	23	**36**	25	23
*% Optimal*	9	**30**	27	21	*Optimal*	6	18	5	6
*% Unknown*	3	6	8	5	*Unknown*	1	1	0	1

### TS 5: Risk perception

**Fig. 4:**
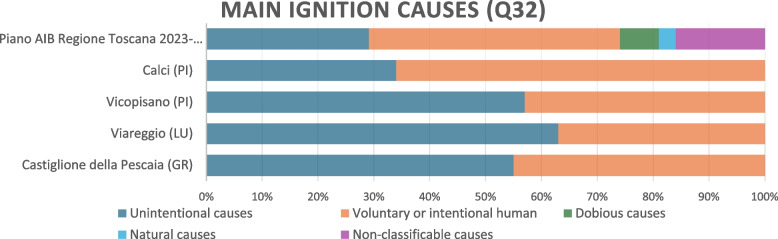
Risk perception. Causes of ignition for wildfires according to the non-experts of the critical areas and comparing with Piano AIB Regione Toscana 2023–2025

By examining Q32, all non-experts believed that the main causes of forest fire ignitions were exclusively due to human behavior: 52% unintentional human causes and 48% voluntary or intentional. A general overestimation of the unintentional causes could be observed (Viareggio for 63%), even with a wide relevance for the voluntary ones (Calci for 66%) (Fig. 4). However, the results of the Tukey test showed that there were no statistically significant differences between the percentages of causes of forest fire ignitions between the “AIB Plan 2023–2025” (real data) and the four selected locations (surveyed perception data, *P* > 0.05 for all comparisons). This suggests that there is a fairly good level of awareness of the causes of forest fires.

**Fig. 5:**
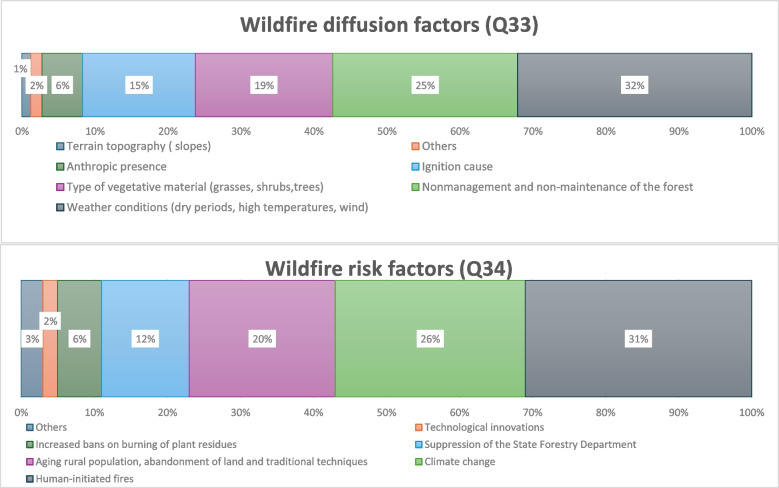
Risk perception. **A** Preferences (in %) for more typical factors of wildfire diffusion, according to the non-expert group only, and considering all critical areas together (Q33). **B** Preferences (in %) for more typical factors of risk for wildfire occurrence, according to the non-expert group only, and considering all critical areas together (Q34)

The wildfire diffusion and risk factors (Q33, Q34) were prioritized by non-experts as shown in Fig. 5A and B. The three factors believed to mostly influence the spread of forest fires were: weather conditions (32%), lack of forest management and maintenance (25%), and the type of vegetative material (19%). Perceived risk factors were uniformly distributed across the four areas, without significant differences in opinions (*χ*^2^ = 28.128, df = 18, *P* = 0.060). Human-initiated fires were considered the main factor leading to increased wildfire risk (31%), followed by climate change (26%), and the population aging and abandonment of inner territories (21%).

**Fig. 6:**
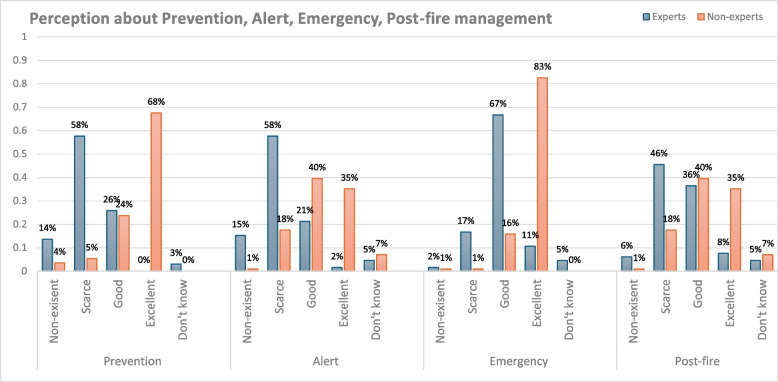
Risk perception. Evaluations of perception (in %) about prevention, alert, emergency, and post-fire phases, comparing experts (blue) and non-experts groups (orange), across all areas

Considering the phases of the Tuscany Region Fire Operational Plan, non-experts (Q35–Q37) expressed more positive opinions than experts (Q17–Q20). In all phases, the statistical analyses revealed significant differences among the groups (FET, *P* ≤ 0.001). The following descriptive statistics explain better. Considering the AIB Prevention intervention, experts observed greater gaps or areas for improvement than non-experts (72% of experts expressed a negative opinion, while 92% of non-experts expressed a positive opinion). In the same way, the Alert phase was considered favorably by the majority of non-experts (75%) and critically by experts (73%). The Emergency phase represented a re-approaching between the two groups, rating it positively (non-experts for the totality, and experts for 78%). Both groups also perceived the post-fire management as Good, despite highlighting a slightly more optimistic view by non-experts. (Fig. 6).

When asked about what made them feel in danger (Q38), almost 50% of non-experts chose “A particular season” and “The sight of wildfire-fighting vehicles in action.” Following, and placed on par (17%), were “Flames hit the neighbor’s house” and “The fire is in action but far away” as reasons for concern, despite evoking two contrasting scenarios. The perception of being in danger during a wildfire varied across the four surveyed locations: Calci (PI), Castiglione della Pescaia (GR), Viareggio (LU), and Vicopisano (PI). A *χ*^2^ Test for Independence was conducted to examine the relationship between location and perceived wildfire risk in different conditions. The test revealed a significant association (*χ*^2^ = 36.068, df = 18, *P* = 0.006916). Post-hoc analyses using pairwise *χ*^2^ tests with Bonferroni correction (1936) indicated that residents of Castiglione della Pescaia reported significantly higher risk perception in scenarios involving “Visible firefighting efforts” and “When a neighbor’s house was affected” compared to the other locations. Conversely, Castiglione della Pescaia showed lower concern “When fires were distant.” Viareggio displayed higher sensitivity to “Alerts,” whereas Vicopisano exhibited a unique concern during “Preventive measures.”

### TS 6: Further remarks

The final section was designed to facilitate participants’ expression of their opinions regarding potential future enhancements to improve wildfire risk management within their specific critical area. The main question (Q31, Q41) was open-ended, and the answers were categorized by twenty subcodes, according to keywords found in the submissions, and then further classified into three main codes, i.e., Public policies and activities (e.g., information, communication, and awareness-raising activities, educational activities, sanctions and controls, policies and responsibility of institutions), Techniques of fuel management (e.g., self-protection, silviculture, firebreaks, prescribed burnings), and Other risk prevention practices and tools (e.g., Training activities, preventive approach, forest communities, modeling and other analysis techniques). Several analyses were conducted for all groups and critical areas, and major results are summarized in the following Table 5, showing, through descriptive statistics, the general prevalence of suggestions in the “Public policies and activities” field (70% non-experts, 82% experts) regardless of the critical area and the group of belonging. To deepen, see the proper sheets (“TS6_Enhancing suggestion”) in the additional files. In the field of “Techniques of fuel management,” “Prescribed burning” was mentioned only by one expert from Calci, which highlighted the need to expand knowledge about this issue and important management practice.

**Table 5:** Further remarks. Responses to the open question, according to the three main codes: in a) percentages of coded further remarks per critical area; in b) percentages of coded further remarks per interviewed group. Data were collected in Tuscany, IT, between April and September 2022

**a) Coded answers per critical area (%)**							
Code/critical area	*Calci* *(n* = 60)		*Castiglione della Pescaia* *(n* = 22)		*Viareggio* *(n* = 30)		*Vicopisano* *(n* = 32)
Public policies and activities	67		86		83		75
Techniques of fuel management	18		9		7		19
Other risk prevention practices and tools	15		5		10		6
**b) Coded answers per interviewed group (%)**							
Code/group		Non-experts (*n* = 79)		Experts (*n* = 65)		Non-experts + experts (*n* = 144)	
*Public policies and activities*		70		82		75	
*Techniques of fuel management*		19		9		15	
*Other risk prevention practices and tools*		11		9		10	

Statistical tests were used to evaluate associations among collected answers, according to the following variables: group (expert or non-expert), location, and code. Data were first aggregated per respondent. Fisher’s exact test and the Kruskal–Wallis tests were applied, but no statistical difference was found among the answers. The results of these tests are reported in Table 6.

**Table 6:** Further remarks. Results from statistical tests (Kruskal–Wallis and Fisher’s exact test), run on the coded answers of the last open question about further remarks. Results showed no significant differences, considering the location (critical area) and the type of respondent (group). Data were collected in Tuscany, IT, between April and September 2022

Kruskal–Wallis *χ*^2^ test		Fisher’s exact test	
*Variables*	*Result*	*Variables*	*Result*
Policies by group	*χ*^2^ = 1.84, df = 1, *P* = 0.17	Group	*P* = 0.758
Management by group	*χ*^2^ = 0.09, df = 1, *P* = 0.77	Critical area	*P* = 0.336
Other by group	*χ*^2^ = 0.74, df = 1, *P* = 0.39		
Policies by critical area	*χ*^2^ = 1.17, df = 3, *P* = 0.76		
Management by critical area	*χ*^2^ = 1.40, df = 3, *P* = 0.71		
Other by critical area	*χ*^2^ = 1.60, df = 3, *P* = 0.66		
Total by group	*χ*^2^ = 0.88, df = 1, *P* = 0.35		
Total by critical area	*χ*^2^ = 1.12, df = 3, *P* = 0.77		

Within the same section, final questions addressing the dissemination of preventive measures, as well as the need for a shift in the wildfire management system from fire suppression towards prevention, revealed a predominantly positive response from interviewees, with 93% of the entire sample (94% non-experts, 92% experts) supporting a more preventive approach (Q40, Q30), even considered by 90% of non-experts a lower-cost strategy compared to suppression (Q39).

## Discussion

### Type of respondent-oriented risk perception

#### Perception within and between the two interviewed groups: similarities, differences, outcomes.

An initial consideration concerns the availability of taking part in the investigation by the two selected groups: the cluster of non-experts was more likely to participate (*n* = 120); indeed, those respondents were almost twice as many as the expert group (*n* = 66). As theorized by Reed (2019), the second group could consider our investigation as something they already manage, often facing it as the last task in the importance of their work, finally a nuisance. On the other hand, non-experts may be more available to participate under certain circumstances (e.g., opportunity for active participation, interest in their needs), still considering the difficulty of including some marginalized groups (e.g., shepherds, people living far from urban centers, women, non-white or migrant people, poor people).

The main objective of identifying gaps between the two groups was achieved, as they often differed in their perception of wildfire risk. A significant gap emerged regarding the prevention system: non-experts’ knowledge about it was shallow, while experts identified numerous existing programs, likely underpromoted. If such programs exist but local communities remain unaware of them or their effectiveness, this gap must be addressed through communication improvement efforts (Cooper et al. 2020; Spano et al. 2021; Alcasena et al. 2022). In many cases, programs need to be funded more, or they have just a little time for their realization, or they have produced little benefits without success in lowering the risk (Daniel et al. 2007; Slovic 2016; Otero et al. 2018). Non-experts expressed concerns primarily about ecological impacts, human losses, and infrastructure damages, reflecting awareness of the ongoing ecological crisis (Pihkala 2020; Vigna et al. 2021b). The study found relatively low levels of concern about the potential economic impacts of wildfires across all areas. This lack of concern was consistent across municipalities, likely reflecting the limited presence of economic sectors directly tied to forest resources or wildfire impacts at both national and local levels. However, the need for a wood market is becoming increasingly evident in light of global decarbonization goals (Ministero delle politiche agricole alimenta (2021)). The only notable economic activity connected to this environment is tourism, which is mainly concentrated in coastal areas. Non-experts generally expressed confidence in the current emergency response system, though experts held a less favorable view of the relationship between communities and other actors. Results from questions on risk perception deepened these opinions, showing that non-experts had a more favorable belief than experts for every phase of the fire prevention management plan, which could indicate a lower perception and underestimation of risk. Non-experts’ perception was also measured through imagery and experience (Martin et al. 2009; Christianson et al. 2013; Champ and Brenkert‐Smith 2016; Spano et al. 2021), seen the importance of the narrative in shaping understanding and perceptions of natural phenomena (Fisher 1987; Duit 1991; Castelló and Montagut 2019), and associated comparisons in wildfire science and management (Paveglio et al. 2019). Historical memory analysis is closely tied to perception shaping imagination and narrative building. This study found stronger recollections associated with larger or more recent wildfires, regardless of their frequency or location. This preference may be linked to media habits, as significant events are generally depicted through high-impact visuals in newspapers or online platforms, making them more memorable. Regarding media habits, the poor evaluation from both groups of respondents highlighted the need to enhance communication tools across all four phases of wildfire management. The findings on preventive communication embodied an urgent issue: while experts rated it positively, non-experts gave a poor evaluation, indicating a communication gap that must be addressed. Broadly, risk perception is shaped by mental models and psychological mechanisms, internalized through social and cultural learning, and influenced by media reports and communication processes (Morgan et al. 2001). The final perception may differ according to the biophysical and social-demographic context, the subject and its experiences, and the object (Daniel et al. 2007; Wachinger et al. 2013). Several gaps in perception were found between experts and non-experts, with non-experts generally underweighting the risk (Meldrum et al. 2015).

The two groups’ suggestions for further remarks were broadly similar, with several exceptions. The “Techniques of fuel management” were scarcely mentioned by either group, which is surprising for experts, but less for non-experts, who likely lack the knowledge or awareness of wildfire safety. Prescribed burning, which refers to the use of fire as a technique to reduce fuels and thus effectively decreases the risk of forest fires (Ascoli et al. 2012; Bonanomi et al. 2022), was mentioned by only one expert. The current management system in Italy often hinders the use of this technique, mainly for cultural reasons. (Bovio and Ascoli 2012; Ascoli and Bovio 2013; Mathews and Malfatti 2024). Prescribed burning stems from the traditional use of fire, controlled by local communities, to achieve various objectives related to the management of the territory.. Fuel management techniques alone may not be sufficient to address the risk in terms of the mitigation efforts required. Therefore, it is also important to consider the adaptation approach, which is mostly influenced by the government policies and depends on the trust between authorities and citizens (Canadas et al. 2023).). The widespread preference for “Policies and public activities” among the majority of respondents clearly highlighted the need for a shift—not only in wildfire management but also in the decision-making process. The question about the shift to a wildfire prevention system was designed to raise the awareness of the respondents rather than to provide supporting statistical data. The point of the question was to get people thinking about the importance of prevention. According to many authors, the system should be reoriented to focus on prevention rather than suppression or emergency response (Daniel et al. 2007; La Mela Veca et al.^ 2024^). It is proven that territories with more active land governance showed lower wildfire impact (Leone and Tedim 2020; Ascoli et al. 2023; Spadoni et al. 2023) and that current policy and governance systems have many failures (Fernandes et al. 2020; Kirschner et al. 2024). In the current global context, research should also act as an intermediary for a just transformation between local communities’ needs and practitioners’ or policy-makers’ interests, by increasing sensitivity to context, representing and legitimating diverse voices, and managing power dynamics (Reed and Rudman 2023). Educational programs were among the most frequently suggested, reflecting widespread demand, despite their relatively low enrollment when offered, likely due to a lower perception of personal danger compared to that of others (Daniel et al. 2007).

### Geographically-oriented risk perception

#### Discussion about the current perception according to the area, involving suggestions in case of a lack or comparison to other territories

The greatest part of the respondents came from the Monte Pisano area (Mathews and Malfatti 2024), the one with the largest and highest number of wildfires, indicating a greater awareness of the topic in that area. Viareggio was the only area where, when asked about the current situation, the two groups were aligned in assessing it as lacking in prevention: little confidence is expressed by both groups. The distinction between *fuoco* (fire) and *incendio* (wildfire) appeared to be rooted among non-experts. Words associated with *incendio* were more markedly negative than those associated with *fuoco*. This probably reflected the idea that wildfire is more often perceived as a destructive and uncontrollable event, whereas fire may have more ambivalent connotations, including useful or symbolic aspects alongside dangers. Controlled fire (*fuoco*) was thus perceived as an essential resource for daily life, symbolizing protection and sustenance; conversely, *incendio* represented a loss of control and a return to natural chaos, emphasizing the duality of fire as both a creative and destructive force in human culture. Significant differences were detected among localities, shaped by local and cultural context. In Calci, a strong sense of local awareness and sensitivity was evident, whereas Castiglione della Pescaia and Vicopisano recorded similar perceptions, suggesting different cultural or experiential perceptions among these communities. These variations in perception have important implications for communication and fire-related emergency management, highlighting the need to tailor messages to the understanding and expectations of each local population. The historical memory analysis revealed notable differences in wildfire events recollection across areas. Viareggio displayed the weakest memory, likely due to the relatively smaller size of burned areas. In contrast, Calci demonstrated the strongest recollection, with the 2018 wildfire being the most mentioned overall, reflecting its lasting impact on the local community. Social memory (Adger et al. 2005) plays a crucial role in gaining valuable experiences to better prepare for future disturbances. Calci’s preparedness was enhanced by its community’s experiences with major wildfires, whereas Viareggio’s limited exposure and social memory hindered similar growth. Nevertheless, even low-impact events should be used as adaptive learning tools to prepare for more severe events (Pausas and Leverkus 2023). Non-experts in Viareggio expressed trust in the coordination system, but this trust was not reciprocated by experts, who assessed the relationship as poor—an obstacle to adaptive learning. This highlights the need to raise awareness, starting with expert training, as trust between communities and agencies is crucial for developing effective policies and management (Slovic 2016; Reed and Rudman 2023). In Viareggio and Castiglione della Pescaia, practitioners showed knowledge and risk perception gaps. Alarmingly, some experts were unaware of or reported the absence of local coordination systems, despite the existence of a regional forest fire prevention and response system. Findings from the risk perception analysis also indicated that location-specific factors significantly shape wildfire risk perception, underscoring the need for tailored communication and intervention strategies. Knowledge exchange is critical to bridging perception gaps, especially in natural hazards (Hermans et al. 2022). Encouraging collaboration among diverse experts and actors through training and learning opportunities can foster new insights and innovations by leveraging complementary perspectives (Tengö et al. 2014).

### Towards an inclusive and interdisciplinary wildfire risk management: the role of participation and social research

Despite requests from the scientific community for a more integrated, inclusive, and adaptive wildfire risk management (Bacciu et al. 2022), rural fire management relies predominantly on technical and instrumental strategies while neglecting the socio-ecological context and local dynamics. As highlighted by this study, people's awareness is strongly related to and influences their preparedness. Areas with a history of wildfire prevention efforts (e.g., Calci and Vicopisano) exhibited higher risk perception, while regions with limited prevention actions (e.g., Viareggio) demonstrated lower preparedness and increased vulnerability (e.g., Massarosa wildfire, 2022). This underscores the need for inclusive, bottom-up strategies that integrate ecological models with social perspectives. The current paradigm, centralized and suppression-focused, has demonstrated its limitations, particularly in complex contexts such as Southern Europe. In the European Mediterranean, fire management remains largely entrusted to public organizations and state agencies, heavily relying on public resources and central authorities (Fernandes et al. 2020). Interdisciplinarity is key in addressing the complexity of relationships between humans, landscapes, and fire regimes. Approaches that integrate ecology, geography, sociology, anthropology, education sciences, and history enable the analysis of biophysical aspects and the social and cultural dynamics that influence the vulnerability and resilience of socio-ecological systems (i.e., Pyrogeography, see Kirschner et al. 2024). Promoting a paradigm shift in Mediterranean landscapes also means recognizing fire as an ecological element while limiting its damage and implementing innovative, nature-based solutions such as controlled grazing, selective thinning, and low-intensity prescribed burns (Lovreglio et al. 2014; Bacciu et al. 2022; Uyttewaal et al. 2023; Ascoli et al. 2023). Stakeholder participation is widely recognized as critical for building resilience at the local level, both in the short and long term (Otero et al. 2018; Lambrechts et al. 2023). A variety of tools with different levels of stakeholder involvement—such as surveys, interviews, focus groups (Kirschner et al. 2024; Chastain and Islar 2024), participant observation, ethnography (Mathews and Malfatti 2024), and participatory workshops—are fundamental for understanding socio-ecological systems, identifying vulnerabilities, and fostering dialogue between academia, institutions, and local communities (Corbetta 2003). Specifically, involving stakeholders in research processes helps reduce information asymmetry, enhance local knowledge, and build shared responsibility and active risk management. This enables a transition from mere consultation to co-creating shared strategies (Bagliani and Dansero 2011; Grifoni et al. 2014). Integrating scientific knowledge with practical experiences while involving local stakeholders is essential for developing tools and processes that can respond accurately and appropriately to the specific challenges of each context (Paveglio et al. 2018).

Active engagement in decision-making processes enables the blending between local and scientific knowledge (Laituri et al. 2023), recovering and strengthening the socio-ecological knowledge specific to a given context, which is at risk of being lost (Mathews and Malfatti 2024). The use of questionnaires in this research was useful to understand the current perception of the studied areas, and encourage different decision-making processes. Future insights could start from the suggested enhancements, focusing on specific places, and using social research methods (interviews, focus groups, etc.) and a participatory process to undertake the actions aimed at enacting an effective decision-making process through the involvement of stakeholders. Moreover, social research techniques and participatory methods enhance community engagement and highlight the specificities of social and environmental contexts and knowledge, promoting the identification of adaptive and shared solutions (Paveglio et al. 2019).

Wildfire mitigation strategies, such as prescribed fire, often conflict with other management paradigms, particularly conservation-related ones (Branca et al. 2020). The survey analyzed in this study showed almost no mention of prescribed burning when interviewees were asked for further management enhancement, suggesting a lack of knowledge or a deliberate ignorance of the tool, probably built on the current normative and cultural system of wildfires. Communication difficulties and administrative barriers, which hinder the implementation of prescribed or controlled fire, further exacerbate the local conflicts and awareness (Ascoli and Bovio 2013). Overall, active participation and interdisciplinarity are promising to effectively address the complexity of socio-ecological systems.

## Conclusion

Our investigation revealed that gaps in wildfire risk perception and management are more pronounced between experts and non-experts than across localities. These gaps can be mitigated through enhanced community engagement and knowledge exchange, reinforcing relationships among territorial stakeholders. This is critical, as wildfires are fundamentally a socio-ecological issue requiring holistic approaches. While acting as an ecological process, wildfires can also serve as catalysts for social organization, particularly in territory management and decision-making.

Encouragingly, both groups of actors acknowledged public action as the primary tool to address wildfire challenges. In our opinion, this could lay the foundation for the activation of participatory processes, directly involving communities. Investigating risk perception and experiences can help uncover these gaps, enabling adaptive strategies and mitigation measures. As expected, wildfire memory and perception were linked to event magnitude: Calci and Viareggio represented two extremes, with Viareggio’s lower perception indicating an opportunity to adapt through increased local awareness. Addressing currently low levels of risk perception, for example, by coupling expert training and the involvement of non-experts in planning and implementation, is essential to bridging these divides. Emphasizing adaptive governance and damage reduction, rather than solely minimizing burned areas, is pivotal. Broad public trust in emergency responses provides a strong foundation for systemic improvements. Acknowledging the human dimension as integral to ecological processes highlights the importance of reintegrating fire into social, economic, and cultural frameworks, fostering proper land management. This implied a growth of the culture of fire, as well as a culture of risk, that is, be prepared to live with the risk, developing collective knowledge about and, first of all, focusing on prevention of the risk itself.

Our study highlights the social dimensions of wildfire risk, the importance of a prevention-based perspective, the value of a transdisciplinary approach in addressing a complex phenomenon, thus highlighting the needs and the significance of the dialogue among different actors and their epistemological frameworks.

### Limits

This exploratory study serves as a starting point for a broader qualitative and empirical investigation needed to deepen knowledge in the region. While representing an initial step toward understanding wildfire risk perception in local communities in Tuscany, it has several limitations. First, the study relies primarily on quantitative data, which, although useful for identifying general trends and differences, do not allow for an in-depth exploration of the social and cultural dynamics influencing the perceptions revealed. Further research combining qualitative and quantitative approaches is necessary to grasp the complex interplay between the social and environmental components of the socio-ecological systems explored.

Consequently, greater critical engagement from the social sciences is essential to better understand and represent the social diversity in each study area and local patterns. Such engagement ensures that local specificities are adequately integrated into risk management strategies, making them more inclusive and targeted. The adopted approach, focused on questionnaires, may have oversimplified certain complexities and underestimated the importance of local context and individual experiences. Additionally, studies exploring the relationship between perception and damage—or potential damage—should incorporate demographic variables and validate whether “concern” and “perception” are consistently interpreted across groups and areas. Future research should deepen the perception differences between experts and non-experts, using tools like focus groups, interviews, and community activities, with a focus on critical areas. These methods allow us to explore more deeply the perceptions, needs, and experiences of the diverse actors involved.

## Supplementary Information

Supplementary Material 1.

Supplementary Material 2.

Supplementary Material 3.

Supplementary Material 4.

## Data Availability

The wildfire dataset used for this study is publicly available at the following link: https://www502.regione.toscana.it/geoscopio/incendiboschivi.html. Some generated datasets are attached as additional files: a context analysis, a list of contacted experts, questionnaires (IT and EN), and an analysis of the last TS. Other datasets used and/or analyzed during the current study are available from the corresponding author upon reasonable request. Clinical trial number: not applicable.
