# Supplementary material for: Wildfire risk perception survey: insights from local communities in Tuscany, Italy
Source: Fire Ecol. 2025 Jun 20;21(1):38. doi: 10.1186/s42408-025-00380-5 (PMC12181119; doi:10.1186/s42408-025-00380-5)
Supplement: Supplementary file 3 — Supplementary Material 3. [file 42408_2025_380_MOESM3_ESM.docx]

# CONTEXT ANALYSIS BY STUDY AREAS

- vegetation description (%woodland, vegetable types, agricultural/woodland, private/public uses) (<https://www.regione.toscana.it/-/geoscopio>)
- socio-economic and demographic description (tourism, firewise or forest community, population, etc.)
- [stakeholders present](https://docs.google.com/spreadsheets/u/0/d/1hp47Gulj0UfPSEZXzT_3jlTO7SjAPGoL2o3TaJoqA4s/edit) (associations, organizations, volunteers, etc.) - land management
- description of the territory (SCIs or protected areas or monumental trees)
- fires (particularly interesting events in recent years, fire history)
- Communication level (see data already processed)
- projects currently underway in the field (European, national, regional, local,..)
- events where questionnaires can be submitted (festivals, AIB anniversaries, institutional events, local assemblies, etc.)

**Grosseto - Castiglione della Pescaia**

- vegetation description (%forest, vegetable types, agricultural/woodland, private/public uses)
  - Area of 2,530 hectares of forest (70%)
    - Major types of vegetation present: pure or mixed pine forests, holm oak forests, sclerophyll forests;
      - Coast: pure or mixed pine forests, sclerophyll forests, evergreen sclerophylls with conifers, holm oaks;
      - Inland: sclerophyllous woods, holm oak forests + a few formations of: evergreen sclerophylls with broad-leaved trees, turkey oaks, chestnut groves, pure or mixed pine forests, sclerophylls with conifers;
      - Close to inhabited areas: pure or mixed pine forests, evergreen sclerophylls with conifers, sclerophyll woods, chestnut groves, holm oaks.
- socio-economic and demographic description (tourism, firewise or forest community, population, etc.)
  - 7148 inhabitants, density of 34.16 inhabitants/km²
  - the construction of a firewise is planned for the Tuscany Region
  - Pineta Scarlino, plan expected news?
- stakeholders present (associations, organizations, volunteers, campsites, etc.) - land management
  - Referent of the Tuscany Region AIB/Forestation
  - Nappi elena – mayor (civil protection)
  - Mazzarello Federico – Deputy Mayor **(**Urban Planning and Territorial Government – State Property – Infrastructure and Roads – Urban Regeneration – Agriculture)
  - Lorenzini susanna – councillor **(**tourism – budget – public works – public space and common good)
  - Mariani isabelle – president of the municipal council **(**sport – associations and volunteering – street furniture and enhancement of the medieval village)
  - [VAB Castiglione della Pescaia](https://www.facebook.com/VAB-Castiglione-della-Pescaia-1384110395052555/)

## [Camping Village Baia Azzurra](https://www.legambienteturismo.it/strutture/italia/toscana/castiglione-della-pescaia/campeggio-villaggio/camping-village-baia-azzurra/)

## [Camping Internazionale Etruria](https://www.legambienteturismo.it/strutture/italia/toscana/castiglione-della-pescaia/campeggio-villaggio/camping-internazionale-etruria/)

- - Legambiente castiglione ([fb](https://www.facebook.com/legambientecastiglione/), [website](http://legambientecastiglione.altervista.org/), telephone 347 990 6955, mail [legambientecastiglione@gmail.com](mailto:legambientecastiglione@gmail.com))
  - [maremma wheels on fire](https://www.facebook.com/maremmawheelsonfire/) (associazione enduro - [airspock73@hotmail.com](mailto:airspock73@hotmail.com))
  - [Maremma Freeride](https://www.maremmafreeride.com/?fbclid=IwAR1VkMi248mkBOadArOKpIktTKgOZIWOvSzIL5vI3VGzMFaQBDbUocgJZp8) (Maremma Cycle Tourism Association, 335 521 4847)
  - [Experience](https://www.experiencemaremma.it/?fbclid=IwAR3e5hAjcTJjJ7xbpeYP1XgZqdoZ91Tmlj5kNUKzvZB0u7GAI3zAoNo5Wqo) Maremma (NGO for the promotion of tourism in the Maremma)
  - [Bagnoli Bike](https://bagnolibike.com) (cycle tourism, enduro, freeride, castiglione)
  - [Uisp Grosseto](http://www.uispgrosseto.it) (Sport, Environment)

## Truffle Hunters Association of the Grosseto Maremma ([fb](https://www.facebook.com/tartufaimaremma/) - telephone 331 951 6836 - [tartufaidimaremma@gmail.com](mailto:tartufaidimaremma@gmail.com))

- - [fridays for future grosseto - maremma](https://www.facebook.com/FridaysForFutureMaremma) (fffgrosseto@gmail.com)
  - Fiab (cycle municipalities), [fiab grosseto](https://www.fiabgrosseto.it/press/news/comuniciclabili-fiab-grosseto-castiglione-della-pescaia-e-massa-marittima-ricevono-da-fiab-le-prime-bandiere-gialle-del-2022)
- description of the territory (SCIs or protected areas or monumental trees, accommodation facilities)
  - Diaccia Botrona Nature Reserve
  - Punta Ala and Isolotto dello Sparviero
  - Roccamare pine forest
  - Tombolo from Castiglion della Pescaia to Marina di Grosseto
  - Scarlino Marshes (on the border with the municipality of Scarlino)
  - Bandite di Scarlino
  - Campsites in pine forests (see stakeholders)
  - Padule Pian d'Alma (pSIC)
  - Poggio Ballone
  - [AIB specific prevention plan on coastal pine forests](https://www.regione.toscana.it/pinete-litoranee-grosseto-e-castiglione-della-pescaia)
- fires (particularly interesting events in recent years, fire history)
- Communication layer (data already processed)
- projects currently underway in the field (European, national, regional, local,..)
  - [MITOMED+ project, Green Beach](https://comune.castiglionedellapescaia.gr.it/green-beach-progetto-mitomed-plus/)
  - Legambiente (blue flags/tourism)
- events where questionnaires can be submitted (festivals, AIB anniversaries, institutional events, local assemblies, etc.) (see Google Calendar)

**Lucca - Viareggio**

- vegetation description (%forest, vegetable types, agricultural/woodland, private/public uses)
  - Total wooded area 802 ha (about 25%)
    - Pure or mixed pine forests of indigenous species 47%
    - Mixed forests of evergreen sclerophylls and conifers 38%
    - Mixed deciduous and coniferous forests 10%
    - Forests dominated by meso-hygrophilous broadleaves 5%


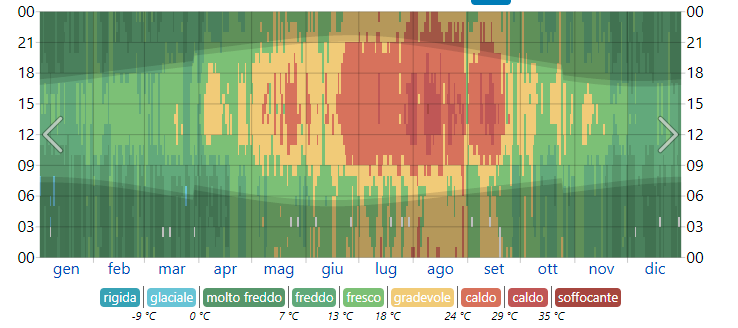


Hourly temperature Viareggio 2020


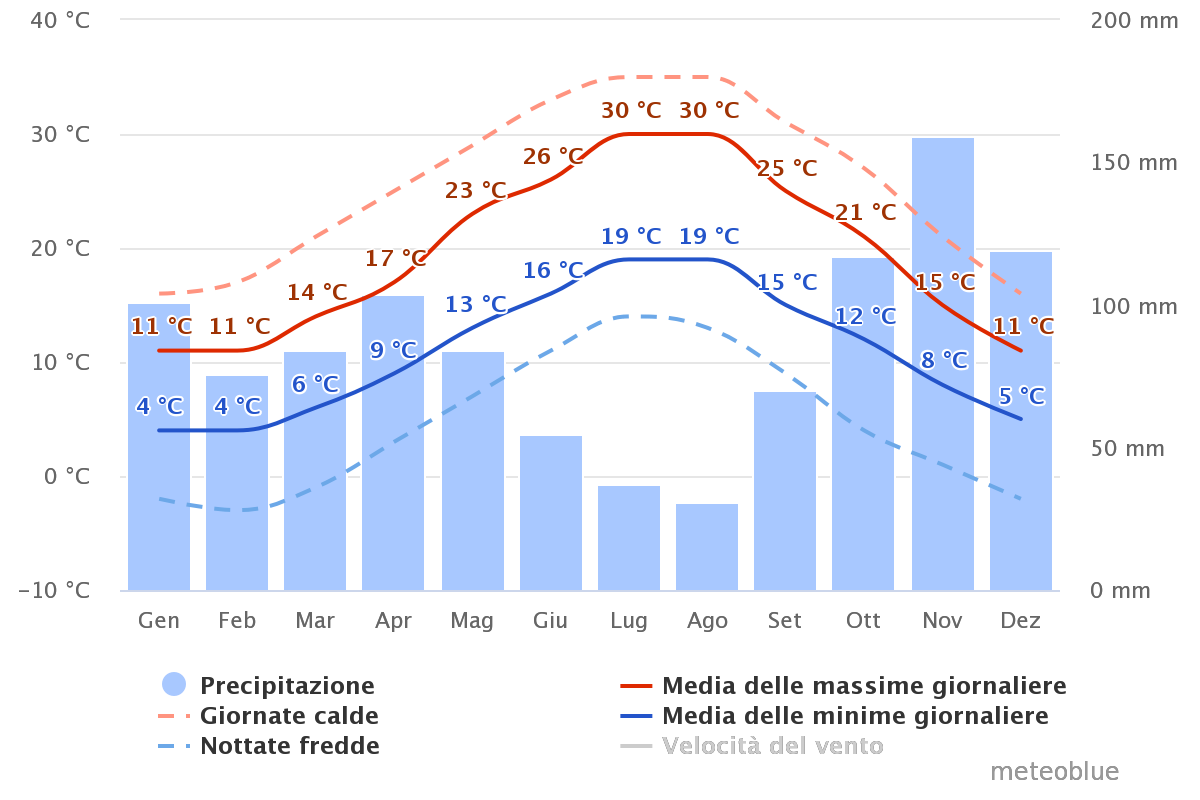


Thermopluviometric Diagram Viareggio

- stakeholders present (associations, organizations, volunteers, campsites, etc.) - land management
  - AGRIAMBIENTE ONLUS: Protection of the natural environment, ecological defense, protection of artistic-monumental heritage and civil protection. E-mail: [agriambiente-lucca@libero.it](mailto:agriambiente-lucca@libero.it)
  - Environmental Guards of Italy ODV (GAD'IT): surveillance activities, protection, protection of the territory, animals and civil protection activities. Email:

[presidenzagaditlucca@gmail.com](mailto:presidenzagaditlucca@gmail.com) , [guardieambientalilucca@pec.it](mailto:guardieambientalilucca@pec.it)

- - LEGAMBIENTE VERSILIA APS: Protection and enhancement of nature and

of the environment, natural resources, collective health, animal and plant species, historical, artistic and cultural heritage, territory and landscape. E-mail: [legambienteversilia@yahoo.it](mailto:legambienteversilia@yahoo.it)

- - G.E.P.A. – Toscana ODV: Carrying out activities of the Zoofile Guards Unit in territorial services, animal protection, education to respect animals and the environment in schools, pet therapy, food collection for kennels and shelters. Email: gepatoscana@hotmail.com
  - Green Cross Viareggio :<http://www.croceverdeviareggio.org/antincendio-boschivo/>
  - ALESSANDRO MECIANI Councillor for Economic Development and Territorial Marketing of the Municipality of Viareggio. Email: [a.meciani@comune.viareggio.lu.it](mailto:a.meciani@comune.viareggio.lu.it)
  - FEDERICO PIERUCCI - Councillor for Urban and Territorial Regeneration: Urban and landscape planning, private construction, hydrogeological and environmental policies, youth policies, participatory processes. mail: [f.pierucci@comune.viareggio.lu.it](mailto:f.pierucci@comune.viareggio.lu.it)
  - GABRIELE TOMEI – Councillor for Welfare: Solidarity economy, social and health policies, housing policies, relationship with volunteering, animal protection, European planning for development strategies, international cooperation. mail: [gabriele.tomei@comune.viareggio.lu.it](mailto:gabriele.tomei@comune.viareggio.lu.it)
  - [Campsite La Pineta](http://www.campinglapineta.com/it/)
  - [Camping Viareggio](https://www.campingviareggio.it/ita/)
  - [Camping Paradiso](https://it-it.facebook.com/campingparadisoviareggio/)
  - [Stacaravan La Dolce Vita](http://stacaravan-la-dolce-vita.viareggiotophotels.com/it/)
  - National Free Hunting Association (3276945817)
  - Bocciodromo asd (3775106284)
- socio-economic and demographic description (tourism, firewise or forest community, population, etc.)
  - 61,183 inhabitants, density 1887 inhabitants/km².
  - Age of inhabitants
    - 0-19 years 15.5%
    - 20-39 years 19.4%
    - 40-59 years 31.4%
    - >60 years 33.7%
  - Tourism data (2021) :
    - **Arrivals** ( the number of customers, Italian and foreign, hosted in accommodation establishments (hotel or complementary) in the period considered):

Italians 112,977

Foreigners 50,816

Tot. 163.793

- - - **Presences** (The number of nights spent by customers in accommodation establishments (hotels or complementary establishments):

Italians 667,695

Foreigners 226,845

Tot. 894.540

- projects currently underway in the field (European, national, regional, local,..)

MITOMED+ Interreg MED "Protection and promotion of the natural and cultural resources of the Mediterranean" "Promoting the development of sustainable and responsible coastal and maritime tourism in the Mediterranean".

- description of the territory (SCIs or protected areas or monumental trees, accommodation facilities)
  - SIR24 ZSC "Macchia Lucchese" (Pineta di Levante)
  - SIR61 ZSC "Coastal Dunes of Torre del Lago"
  - SIR25 ZSC "Lago di Massaciuccoli".
  - Pineta di Ponente Park in Viareggio
  - Migliarino San Rossore and Massaciuccoli Park
  - Monumental trees : 01/L833/LU/09 Quercus ilex Torre del Lago

**Pisa - Calci**

- socio-economic and demographic description (tourism, firewise or forest community, population, etc.)
  - inhabitants 6,374, density 253,24 inhabitants/km²
  - One of the first Firewise communities born in Italy
  - average age 47.1
  - foreign population 3.94%
  - Population age
    - 15.39% < 17 years old
    - 18 < 26.3% < 44
    - 45 < 32.42% < 64
    - 25.88 > 65 years old

- projects currently underway in the field (European, national, regional, local,..)
  - SUCCESS project within the Interreg Italy-France Maritime Programme 2014-2020.
  - CALCI 2024 PROJECT
- stakeholders present (associations, organizations, volunteers, etc.) - land management
- Gea S.r.l. (in liquidation) integrated management of public waste disposal services, sewerage network management and drinking water cycle
- Geofor: carries out waste management activities and tries to create solutions for the correct management of the waste cycle at the lowest possible socio-environmental cost.
- RetiAmbiente S.p.A. carrying out the integrated urban waste management service in the optimal territorial area "Toscana Costa".
- Toscana Energia Spa
- <https://www.toscanaenergia.eu/it/informazioni-ambientali/>
- [Tuscan Water Authority - ATO 2](http://autoritaidricatoscana.trasparenza-valutazione-merito.it/web/trasparenza/menu-trasparenza)
- Rivers and Ditches ConsortiumThe Reclamation Consortium guarantees a service for the protection and regulation of plain and hill waters to improve the conditions of the territory and safeguard it
- AGRITURISTICA DEL LUNGOMONTE PISANO SOC, COOP. VA AGRICOLA
- The new Limonaia - Association for the dissemination of scientific, technological culture and the various branches of knowledge
- description of the territory (SCIs or protected areas or monumental trees)

Since 2003, [the Valle del Lato has been](https://it.wikipedia.org/wiki/2003) a [protected natural area of local interest](https://it.wikipedia.org/wiki/Area_naturale_protetta_di_interesse_locale) in [the province of Pisa](https://it.wikipedia.org/wiki/Provincia_di_Pisa) belonging to the municipality of [Calci](https://it.wikipedia.org/wiki/Calci), located in the central part of [Monte Pisano](https://it.wikipedia.org/wiki/Monte_Pisano).

The Sughera, a tree of quercus suber planted before 1880.

- fires (particularly interesting events in recent years, fire history)

Fire in the Pisan Mountains in 2018, about 1000 hectares of forest burned.

- vegetation description (%forest, vegetable types, agricultural/woodland, private/public uses)

Vegetation cover >50%, with a prevalence of Mediterranean pine forests and woods with a prevalence of chestnut. There are also mixed coniferous and deciduous forests and dense shrubs in smaller quantities.

Land use is mostly made up of semi-natural environments and wooded areas (>60%), the rest is characterized by a large presence of agricultural land and the remaining artificial surfaces.

**Pisa - Vicopisano**

**Consortium 1 Northern Tuscany FOR SOIL RECLAMATION AND PROTECTION**

Telephone: 0584/43.991

Email: info@cbtoscananord.it

PEC: protocollo@pec.cbtoscananord.it

**Sito :** [**https://www.cbtoscananord.it/**](https://www.cbtoscananord.it/)

**Association of Social Promotion of Walking Gods**

The association was founded in 2015 to imagine and carry out activities in nature (trekking, etc.)

Saturday 04- Sunday 19- Saturday 2 Walks in the city

Info and contacts

Secretary: Maura

tel. 347 1493723

info@camminanti.it

**Gruppo Volontari Antincendio F.lli Del Moro Vicopisano**

<https://www.facebook.com/gvafllidelmoro/>

**Vico Verde**

Environmental protection and urban decorum

<https://www.facebook.com/vico.verde/>

**The Acorn Forest**

It deals with raising awareness for children and adolescents on the theme of the forest

<https://m.facebook.com/asilonelboscovico/about/>

**Circolo ARCI L'Ortaccio**

The main purpose of the Association is to promote sociality, solidarity, participation and the development of a sense of community, contributing to the cultural and civil growth of its members, as well as of the entire community.

**Il Frantoio di Vicopisano, a farm**

[**http://www.vicopisanolio.it/index-lingua-1.html**](http://www.vicopisanolio.it/index-lingua-1.html)

**"Roots for the future" project:** About 700 new trees planted in the area; the Mayor Matteo Ferrucci and the Councilor for Youth Policies, Juri Filippi, present the "Roots for the future" project.

**Centro Legno Ambiente Società Cooperativa Arl**

Soil protection, green management, road works, emergency response, naturalistic engineering

**Delca Energy**

Energy recovery, circular economy, reduction of environmental impact

**Calci and Vicopisano**

**Establishment of an Interregional Structural Plan** (agreement signed by the

respective Mayors on 12 March 2020), with the stipulation of the Regional Territorial Address Plan (PIT) and the Provincial Territorial Coordination Plan (PTC).

**CIPC Monte Pisano** (Lead Municipality of the association between the Municipality of Buti, the Municipality of Calci and the Municipality of Vicopisano);

- Update of the Intermunicipal Civil Protection Plan in Emergency

- Management of Intermunicipal CIPC Monte Pisano availability, weather alert system

- Planning and implementation of annual civil protection exercises

- Management of local operations room functionality at the AIB logistics center in Caprona

- Preparation of safety plans for specific events with the support of the AIB structure

- Exceptional situations of particular public safety risk (Monte Serra Ferragosto Operational Plan - Interface Plans)

- Agreement with the Intermunicipal Civil Protection Centre

Office Manager: Ing. Claudia Marchetti

Operational Unit: Dr. Geologist Silvia Lorenzoni

Number of reports during the CESI opening period (events in progress or red alert):Tel. 050939563

**CRED:** The Zonal Conference for education and instruction of the Pisa area is formed by the Mayor, or his delegated Councilor, of the Municipalities of Calci, Vicopisano, Vecchiano, San Giuliano Terme, Cascina and Pisa.

Provides support to the Conference for planning, design, management and

implementation of interventions and area activities aimed at children and young people of age

drain. The issues it deals with are: the fight against early school leaving, the inclusion of disability, intercultural inclusion, the development of intelligences and any other activity aimed at

personalize and promote the success of the educational/training intervention. It also deals with:

sizing of the school network and planning of the educational offer,

school support, formal education of young people and adolescents, lifelong learning and

adult education.

**Feronia,** for excursions and events (also for children), from 13 June to 29 July and from 22 August to 9 September (Mon-Fri), from 8.00/9.00 to 16.00/16.30, in Calci, nature camp for children.

**Vegetation description**

Vegetation cover mainly of pure or mixed pine forests of indigenous species, to a lesser extent there are also holm oak forests. Large presence of dense shrubs. Few mixed coniferous and deciduous forests.

About 40% of the total area is wooded.

About 20% is arable land.

Fewer permanent crops (for the most part olive groves, some in the process of abandonment, but also vineyards).
